# Oral administration of a probiotic *Lactobacillus *modulates cytokine production and TLR expression improving the immune response against *Salmonella enterica *serovar Typhimurium infection in mice

**DOI:** 10.1186/1471-2180-11-177

**Published:** 2011-08-03

**Authors:** Natalia A Castillo, Gabriela Perdigón, Alejandra de Moreno de LeBlanc

**Affiliations:** 1 Centro de Referencia para Lactobacilos (CERELA-CONICET). Chacabuco 145. San Miguel de Tucumán. Argentina; 2 Cátedra de Inmunología, Facultad de Bioquimíca, Química y Farmacia, Universidad Nacional de Tucumán, Argentina

## Abstract

**Background:**

Diarrheal infections caused by *Salmonella*, are one of the major causes of childhood morbidity and mortality in developing countries. *Salmonella *causes various diseases that range from mild gastroenteritis to enteric fever, depending on the serovar involved, infective dose, species, age and immune status of the host. Probiotics are proposed as an attractive alternative possibility in the prevention against this pathogen infection. Previously we demonstrated that continuous *Lactobacillus casei *CRL 431 administration to BALB/c mice before and after challenge with *Salmonella enterica *serovar Typhimurium (*S*. Typhimurium) decreased the severity of *Salmonella *infection. The aim of the present work was to deep into the knowledge about how this probiotic bacterium exerts its effect, by assessing its impact on the expression and secretion of pro-inflammatory (TNFα, IFNγ) and anti-inflammatory (IL-10) cytokines in the inductor and effector sites of the gut immune response, and analyzing toll-like receptor (TLR2, TLR4, TLR5 and TLR9) expressions in both healthy and infected mice.

**Results:**

Probiotic administration to healthy mice increased the expression of TLR2, TLR4 and TLR9 and improved the production and secretion of TNFα, IFNγ and IL-10 in the inductor sites of the gut immune response (Peyer's patches). Post infection, the continuous probiotic administration, before and after *Salmonella *challenge, protected the host by modulating the inflammatory response, mainly in the immune effector site of the gut, decreasing TNFα and increasing IFNγ, IL-6 and IL-10 production in the lamina propria of the small intestine.

**Conclusions:**

The oral administration of *L. casei *CRL 431 induces variations in the cytokine profile and in the TLRs expression previous and also after the challenge with *S*. Typhimurium. These changes show some of the immune mechanisms implicated in the protective effect of this probiotic strain against *S*. Typhimurium, providing an alternative way to reduce the severity of the infection.

## Background

Diarrheal infections caused by bacterial enteric pathogens including *Salmonella*, are one of the major causes of childhood morbidity and mortality in developing countries [1]. *Salmonella enterica *serovar Typhimurium (*S*. Typhimurium) is an intracellular Gram-negative bacterium characterized by its ability to survive and replicate within eukaryotic host cells, particularly epithelial cells and macrophages. In humans, while *Salmonella enterica *serovar Typhi typically causes severe or sometimes lethal systemic illness called "Typhoid Fever", *Salmonella *Typhimurium is associated with self limiting gastroenteritis and requires treatment only in immunocompromised patients. *S*. Typhimurium develops in mice an infection with the same pathogenesis and clinical manifestations than *S*. Typhi in humans thus, this mouse model is useful for the study of this disease [2].

The intestine harbours trillions of commensal bacteria that participate in digestive functions and help to protect the host from the aggression of several enteropathogens [3]. The beneficial effects of the microbiota on the host immune system have allowed the proposal to use some non pathogenic bacteria, such as probiotics in improving animal health and protection against infectious agents [4]. Probiotics have been shown to influence both innate and adaptive immunity through direct contact with epithelial and immune cells, or by their ability to modify the composition and activity of the gut microbiota. They exert their protective effects by multiple immune and non immune mechanisms [5], i.e., exerting direct antimicrobial activity against pathogens [6], increasing phagocytosis [7], modifying cytokine production by different cell populations [8-10] or enhancing IgA production [11]. One of the principal mechanisms of protection against gastroenteric infections by probiotics is via modulation of pro-inflammatory (like IFNγ and TNFα) and anti-inflammatory (IL-10) cytokines, but the pathways and cells involved in this mechanisms are not clear yet [12]. It is a fact that not all microorganisms have the same effect on the host, and that probiotic properties are strain and host specific. In this sense, it is not possible to extrapolate the effects found with one probiotic strain to another, or its effect against a specific pathogen to other pathogen [13].

*L. casei *CRL 431 is a probiotic bacterium and its effects on the gut immune cells have been extensively studied. In a previous work, the effect of *L. casei *CRL 431 in the prevention of *S*. Typhimurium infection in BALB/c mice was evaluated. It was demonstrated that 7 days of *L. casei *CRL 431 administration before *S*. Typhimurium infection decreased its severity. The continuous probiotic administration (before and after infection) diminished the pathogen counts in the intestine as well as its spread outside this organ. The probiotic administration decreased the neutrophil infiltration with the consequent diminution of intestinal inflammation; activated the macrophage phagocytic capacity in Peyer's patches, spleen and peritoneum; and increased the number of IgA(+) cells in the lamina propria of the small intestine which was correlated with increased release of s-IgA specific against the pathogen in the intestinal fluids [7].

The aim of the present work was to deep into the knowledge about how the probiotic bacterium *L. casei *CRL 431 exerts its protective effect against *S*. Typhimurium infection, by assessing the impact of this probiotic strain on the cytokine profile (expression and secretion) and in the expression of different Toll-like receptors (TLRs) in the inductor and effector sites of the immune response in the small intestine, in both healthy and infected animals.

## Results

### Effect of *L. casei *CRL 431 administration on the cytokine producing cells isolated from Peyer's patches in animals non infected or infected with *Salmonella*

Healthy mice that received the probiotic during 7 days (*Lc *group) and mice non-treated with *L. casei *CRL431, but challenged with *Salmonella *(infection control, *S *group) stimulated the production of TNFα and IFNγ by the immune cells of the Peyer's patches, compared to non-treated and non-infected mice (untreated control, C) (Table 1). These cytokine producing cells increased significantly (p < 0.01) 7days post challenge in the mice fed continuously (before and after infection) with the probiotic strain (*Lc-S-Lc *group), compared to the infection control (*S *group). No significant differences with the infection control (*S *group) were observed in the number of TNFα (+) cells isolated from mice that stopped probiotic administration after infection (*Lc*-*S *group), while these last group showed significantly (p < 0.01) decreased number of IFNγ (+) cells compared to the other two infected groups (*Lc-S-Lc *and *S*). The analysis of IL-10 producer cells showed that 7 days of probiotic administration (*Lc *group) and also *Salmonella *challenge (*S *group) increased significantly (p < 0.01) the number of these cells compared to the untreated control (C group). Seven days after infection, both groups administered *L. casei *CRL 431 decreased the number of IL-10 (+) cells to values similar to C group (Table 1).

**Table 1:** Cytokine producing cells isolated from Peyer's patches of mice untreated or treated with *L. casei *CRL 431 previous and post challenge with *S*. Typhimurium

Experimental groups	N° of cytokine secreting cells		
	
	TNFα	IFNγ	IL-10
C	10 ± 4^a ^	13 ± 3^ad^	12 ± 3^a^
*Lc*	24 ± 9^b^	20 ± 3^b^	17 ± 1^b^
*S*	26 ± 4^b^	16 ± 1^ab^	22 ± 6^b^
*Lc*-*S*	36 ± 12^b^	12 ± 2^d^	9 ± 4^a^
*Lc*-*S-Lc*	75 ± 4^c^	41 ± 10^c^	13 ± 5^a,b^

Cytokine producing cells were analyzed by immunocytochemistry in mononuclear cells isolated from Peyer's patches of mice at 2 time points: the day of the infection (basal data) for the untreated control (C) and for mice given *L. casei *CRL 431 during 7 days (*Lc*), and 7 days post infection for infection control (*S*), mice given probiotic 7 days before the infection (*Lc-S*), and mice given continuously probiotic, before and after infection (*Lc-S-Lc*). Results for healthy mice obtained the same day of the infected animals were not added because there were not significant differences compared to the basal data. Results are expressed as the means ± SD of the total number of positive cells per 2 × 10^4 ^counted cells at 1 000X magnification. Means for each value without a common letter differ significantly (P < 0.01).

### Measurement of cytokines released by immune cells isolated from Peyer's patches of mice untreated or treated with the probiotic strain previous and post infection

Cells isolated from Peyer's patches of healthy mice fed 7 days with *L. casei *CRL 431 (*Lc *group) increased significantly (p < 0.01) the release of IFNγ and IL-10 compared to the untreated control (C group). Seven days after infection, the cells from the infection control group (*S*) increased significantly (p < 0.01) the release of IFNγ and TNFα, compared to the untreated control (C). However, at this time point, the IFNγ levels in the culture supernatant of cells isolated from the two groups fed with the probiotic strain (*Lc*-*S *and *Lc*-*S-Lc *groups) decreased significantly (p < 0.01) compared to the infection control (*S*). The concentration of this cytokine from *Lc-S-Lc *group was similar to those obtained from healthy mice fed with *L. casei *(*Lc *group). The production of TNFα did not show significant differences (p < 0.01) in all the groups after *Salmonella *infection. Seven days after infection, the cells isolated from *S *and *Lc-S *groups showed similar releases of IL-10, without significant differences compared to healthy mice (C and *Lc *groups). Continuous probiotic administration before and after infection decreased significantly (p < 0.01) the IL-10 release by the Peyer's patches mononuclear cells compared to the other infected groups, and the values were similar to those obtained from cells of the untreated control (C) (Table 2).

**Table 2:** Effect of *L. casei *CRL 431 administration on the cytokines released in cultures of immune cells isolated from Peyer's patches of mice untreated, treated and infected with *S*. Typhimurium

Experimental groups	Cytokine concentration (pg/ml)		
	
	TNFα	IFNγ	IL-10
C	203 ± 32^a^	139 ± 83^a^	65 ± 13^ac ^
*Lc*	257 ± 55^ac^	1175 ± 563^bc^	187 ± 91^b^
*S*	336 ± 90^bcd^	1384 ± 74^c^	102 ± 42^ab ^
*Lc*-*S*	328 ± 4^b^	148 ± 86^a^	102 ± 24^ab ^
*Lc*-*S-Lc*	432 ± 20^d^	592 ± 40^b^	34 ± 18^c ^

The concentration of different cytokines were evaluated in supernatant of cultures of cells isolated from Peyer's patches of mice at 2 time points: the day of the infection (basal data) for the untreated control (C) and for mice given *L. casei *CRL 431 during 7 days (*Lc*), and 7 days post infection for infection control (*S*), mice given probiotic 7 days before the infection (*Lc-S*) and mice given continuously probiotic, before and after infection (*Lc-S-Lc*). Cytokine concentration in the cell culture supernatants after 24 h of incubation was determined by ELISA. Results are expressed as the means ± SD of the concentrations of each cytokine released into the supernatant (pg/ml). Means for each cytokine without a common letter differ significantly (P < 0.01).

### Effect of *L. casei *CRL 431 consumption on the cytokine producing cells in the lamina propria of the small intestine in healthy and infected mice

**Figure 1:**
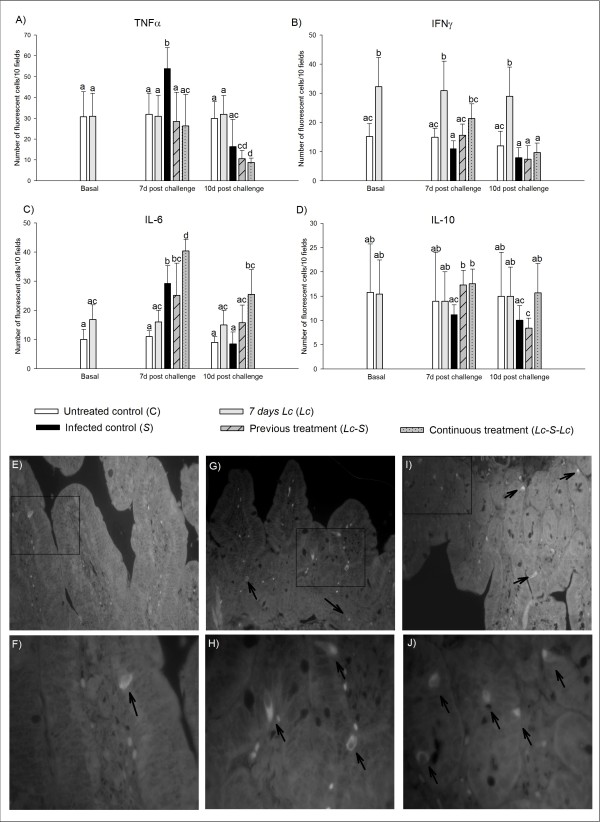
**Determination of cytokine (+) cells in the small intestine tissues**. Positive cells were counted in histological sections from small intestine of mice fed 7 d with *L. casei *CRL 431 previous challenge with S. Typhimurium (*Lc-S*), and mice fed continuously (before and after infection) with the probiotic bacteria (*Lc-S-Lc*), compared to the infection control (*S*). Tissues from healthy mice fed or not with *L. casei *(*Lc *and C groups, respectively) were also analyzed. The samples were obtained the day of the infection (basal data) for *Lc *and C groups, and 7 and 10 days post challenge for all the groups. Representative microphotographs show the differences observed between C group (E and F), *S *group (G and H), and *Lc-S-Lc *group (I and J) in the number of IL-6 (+) cells (arrows), 7 days post challenge. The microphotographs E, G and I were obtained at 400× while F, H and J were taken at 1 000X. A difference of 1 cell at 1000× is related with 10 cells of difference in the final result. Means for each value without a common letter differ significantly (P < 0.01).

The results obtained in the basal samples, before *S*. Typhimurium challenge, showed that the number of IFNγ (+) cells increased significantly (p < 0.01) in the mice given probiotic during 7 days compared with the untreated control (32 ± 10 cells/10 fields vs. 15 ± 6 cells/10 fields Figure 1B). At this time point, TNFα, IL-6 and IL-10 positive cells remained similar in both experimental groups (Figure 1A, C and 1D). TNFα (+) cells were significantly (p < 0.01) increased in the infection control group (*S*) (54 ± 10 cells/10 fields) 7 days post infection, compared with the basal data (31 ± 12 cells/10 fields and 31 ± 11 cells/10 fields for C and *Lc *groups, respectively). Ten days post *S*. Typhimurium infection, the number of cells positive for this cytokine decreased in all the groups challenged, and the decreases in the treated groups were significant (p < 0.01) compared to the basal samples (11 ± 4 cells/10 fields and 9 ± 2 cells/10 fields, for *Lc*-*S *and *Lc*-*S-Lc*, respectively, Figure 1A). Seven days post challenge, the continuous probiotic administration (*Lc*-*S-Lc *group) maintained the number of IFNγ (+) cells (21 ± 5 cells/10 fields) similar to the basal data, being this number significantly higher (p < 0.01) than the observed in the *S *group at the same time point (11 ± 4 cells/10 fields). Ten days post challenge the number of IFNγ (+) cells significantly decreased (p < 0.01) in the *Lc*-*S-Lc *group, and no significant changes for this cytokine were observed between the three infected groups and the untreated control (C) (Figure 1B). The number of IL-6 (+) cells was significantly increased (p < 0.01) in the three groups challenged with the pathogen 7 days post infection, compared to the untreated control group (C). At this time point, the *Lc*-*S-Lc *group also showed a significant increase (p < 0.01) of IL-6 (+) cells compared to all the groups. At day 10 post-challenge, the *Lc-S-Lc *group maintained a number of IL-6+ cells higher than both control groups (C and *S*, Figure 1C). Seven days post challenge, the two groups fed with the probiotic (*Lc*-*S *and *Lc*-*S-Lc*) showed significant (p < 0.01) increases of IL-10 (+) cells compared to *S *group. No significant differences were observed 10 days post infection in the different experimental groups (Figure 1D).

### Cytokine profile on the small intestinal fluid

**Figure 2:**
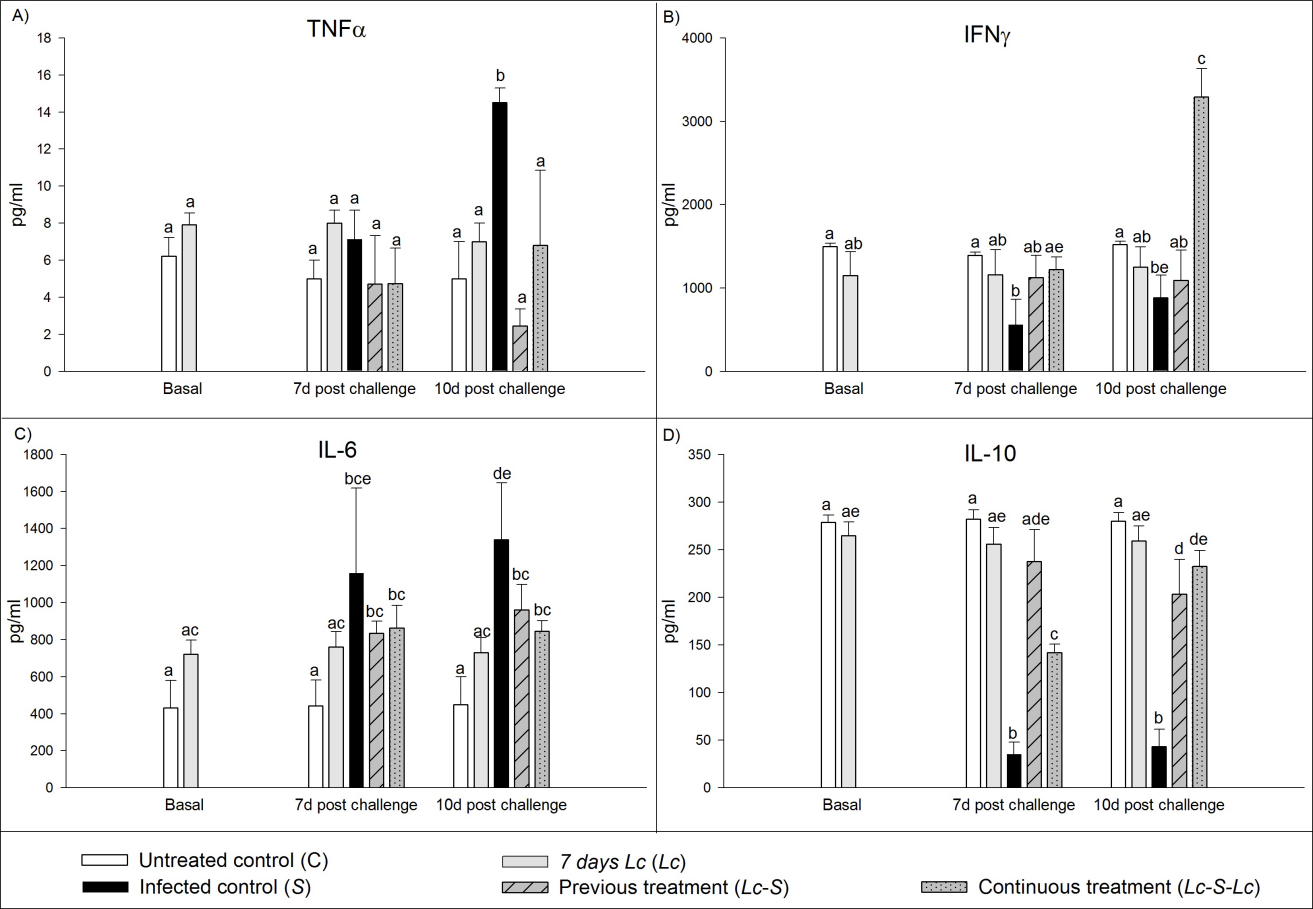
**Determination of the concentration of TNFα, IFNγ IL-10 and IL-6 in intestinal fluid by ELISA**. The samples were taken before the infection for the untreated (C) and *L. casei *CRL 431*(Lc*) groups, and 7 and 10 days post challenge for all the experimental groups. The results were expressed as the means ± SD of the concentration of each cytokine in pg/ml. Means for each value without a common letter differ significantly (P < 0.01).

In the basal sample, after 7 days of feeding, the group *Lc *showed similar levels of TNFα, IFNγ, IL-6 and IL-10 released to the intestinal lumen than the untreated control (Figure 2A, B, C and 2D). The groups *Lc*-*S *and *Lc*-*S-Lc *maintained TNFα concentration in the intestinal fluid similar to basal groups in both samples, 7 and 10 days post challenge; while the release of TNFα was significantly increased (p < 0.01) in mice from *S *group compared to basal samples, 10 days post challenge (Figure 2A). IFNγ levels were significantly higher (p < 0.01) in mice administered continuously with the probiotic (*Lc*-*S-Lc*) compared to the infection control group (*S*) for 7 and 10 days post challenge (Figure 2B). The *Lc-S *and *Lc-S-Lc *groups maintained IL-6 levels in the intestinal fluid similar to *Lc *group, 7 and 10 days post challenge. Nevertheless IL-6 release in *S *group was significantly increased (p < 0.01) 7 days post challenge compared to the untreated control (C), and this levels remained high 10 days post challenge (Figure 2C). IL-10 concentration was significantly increased (p < 0.01) in *Lc*-*S *and *Lc-S-Lc *groups compared to *S *group, for 7 and 10 days post-infection (Figure 2D).

### Effect of probiotic administration and S. Typhimurium infection on TLR2, TLR4, TLR5 and TLR9 expression in the lamina propria of the small intestine

**Figure 3:**
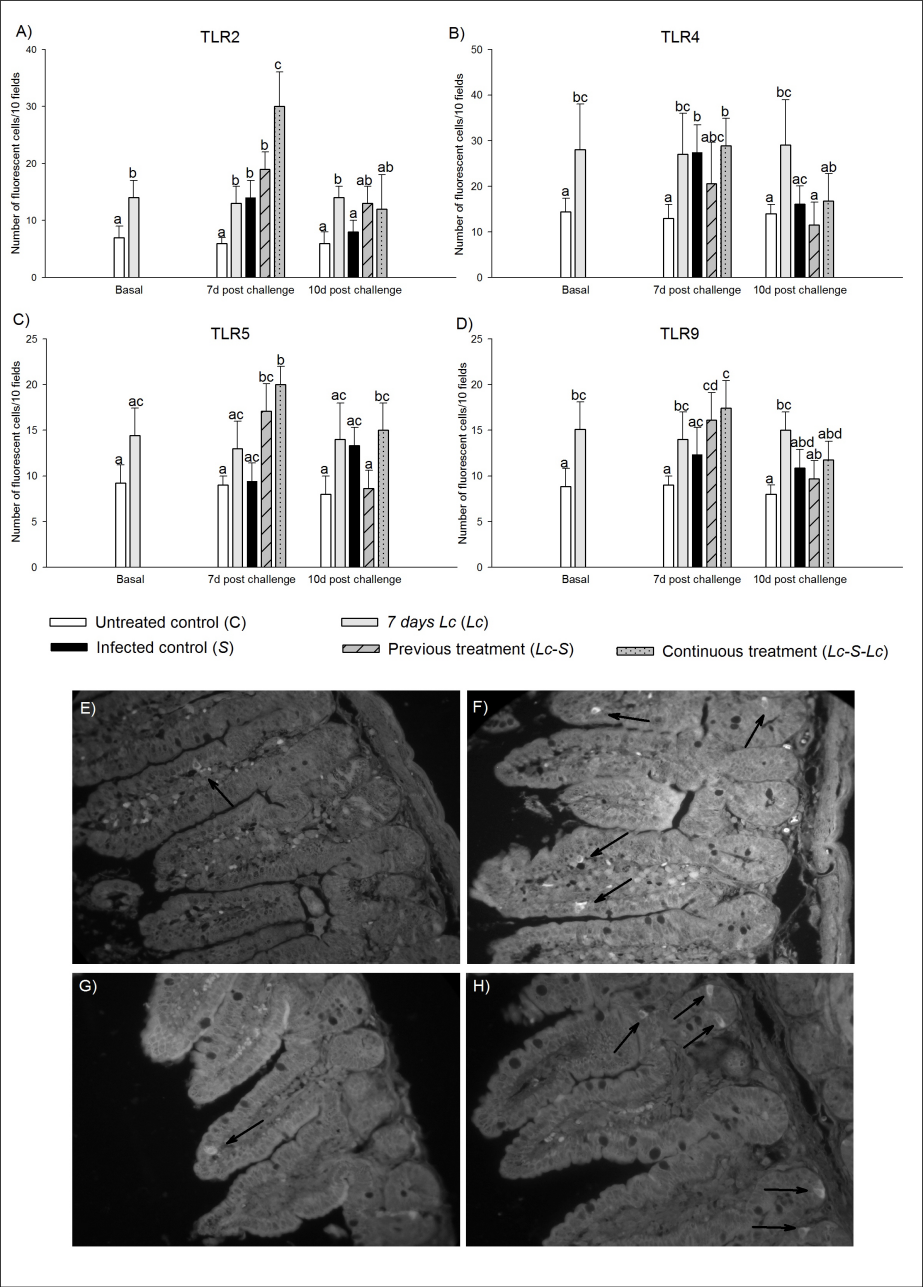
**Determination of TLRs (+) cells in histological sections of small intestine**. The samples were obtained before the infection for the untreated control (C) and healthy mice given *L. casei *CRL431 (*Lc *group), and 7 and 10 days post challenge for all experimental groups. The number of fluorescent cells was counted in 30 fields of vision at 1 000X of magnification and the results were expressed as the number of positive cells counted per 10 fields. The microphotographs (400×) F and H show the increases of TLR2+ and TLR4+ cells, respectively (fluorescent cells) in mice from *Lc *group compared to the untreated control (C group: E for TLR2 and G for TLR4). Means for each value without a common letter differ significantly (P < 0.01).

*L. casei *CRL 431 administration to healthy mice (*Lc*) increased the expression of all the TLRs analyzed compared to the untreated control (C) (Figure 3). Seven days post infection, the mice that received continuously *L. casei *CRL 431 (*Lc*-*S-Lc *group) showed a significant (p < 0.01) increase of TLR2 (+) and TLR5 (+) cells (30 ± 10 cells/10 fields and 18 ± 2 cells/10 fields, for TLR2 and TLR5 respectively) compared to *S *group (14 ± 5 cells/10 fields and 9 ± 2 cells/10 fields, respectively) (Figure 3A and 3C). At this time point, TLR9 (+) cells increased significantly (p < 0.01) in both treated groups (*Lc*-*S *and *Lc*-*S-Lc*), compared to the untreated control (C) (Figure 3D). TLR4 (+) cells increased significantly (p < 0.01) in the infection control group (*S*) and in mice fed continuously with the probiotic strain (*Lc*-*S-Lc*) compared to the untreated control (C), (Figure 3B). For 10 days post challenge, TLR2, TLR4 and TLR9 (+) cells of mice from infected groups (*S, Lc-S *and *Lc-S-Lc*) showed values similar to the untreated control (C), (Figure 3A, B and 3D). For TLR5 the mice from the group *Lc-S-Lc *maintained significantly increased (p < 0.01) the expression of this receptor in comparison with the untreated control (C), (Figure 3C).

## Discussion

A previous work demonstrated that *L. casei *CRL 431 administration induced activation of the immune cells associated to the small intestine of mice that received the probiotic strain [4]. We also observed that this probiotic strain decreased the severity of *S*. Typhimurium infection in a mouse model, showing the continuous administration, the best effect. Continous probiotic administration decreased the mortality percentage (ten times) and the CFU/g of *Salmonella *in liver, spleen and large intestine for 7 and 10 days post- infection [7]. In the present work, some immune mechanisms by which *L. casei *CRL 431 administration exerts its protective effect against *Salmonella *infection were analyzed, as the intestinal cytokine profile in the inductor (Peyer's patches) and effector sites (lamina propria) of the gut immune response. The modulation of TLRs expressions was also determined in the small intestine tissues.

Previous to the infection, analyzing the mononuclear cells isolated from Peyer's patches, it was observed that mice fed 7 days with *L. casei *CRL 431 significantly increased cytokines expression and also the release of IFNγ and IL-10 by these cells. The production of cytokines in Peyer's patches was maintained without significant differences in healthy animals that received the probiotic strain (*Lc*) during all the experiment. These cytokines were also studied 7 days post infection and it was observed that mice from infection control group (*S*) and the group fed continuously with the probiotic strain maintained increased expression of both TNFα and IFNγ in the cells isolated from Peyer's patches. Nevertheless, the release of IFNγ from these cell cultures was significantly higher in the infection control (*S*) than in the mice given probiotic (*Lc*-*S*-*Lc *group). The increases of these cytokines in Peyer's patches are important because they constitute the main inductor site for mucosal immune response. In *S*. Typhimurium infection, this site is one of the pathways that *Salmonella *uses to invade the host, although *Salmonella *infection can also occur through the intestinal epithelial cells along the small intestine [14]. Therefore post infection, we also focused on the cytokine expression in cells from the lamina propria of the small intestine and the cytokines secretion into the intestinal lumen, due to this is the effector site of the gut immune response (Figure 1 and 2). TNFα is a pro-inflammatory cytokine that induces activation and recruitment of neutrophils involved in local inflammatory processes, and produces intestinal epithelial barrier dysfunction, contributing to the entry and colonization of pathogenic bacteria usually excluded from the subepithelial mucosa [15-17]. Seven days post infection, the probiotic administration (*L*c-*S *and *Lc*-*S*-*Lc *grups) was able to maintain TNFα production in the lamina propria of the small intestine and its secretion to the intestinal fluid similar to the observed in the non infected groups (*C *and *Lc *groups). These values showed a tendency to decrease 10 days post challenge. In contrast, the infection control group significantly increased TNFα expression 7 days post challenge as well as its secretion 10 days post infection (Figure 2). The TNFα modulation by probiotic administration could be related with the lesser polymorphonuclear infiltration and inflammation degree in the lamina propria observed previously [7]. Otherwise, the positive cells for this cytokine and its release from these cells were increased in Peyer's patches when the mice received continuously the probiotic strain compared to the untreated control (C). These increments could be related with the high number of activated macrophages present in these sites, suggesting that TNFα is required in the inductor site to maintain the immune response against *Salmonella *(Tables 1 and 2). IFNγ is implicated in the immune activation by probiotic bacteria and fermented milks. It contributes in the activation of macrophages to promote the effective killing of pathogens that can survive within them. In our model, the number of IFNγ (+) cells in small intestinal tissues was significantly lower in the group of mice from the infection control group (*S*) than in the group of mice given continuously *L. casei *CRL 431, which maintained the number of these positive cells similar to the *Lc *group (Figure 1B). As regard to the release of IFNγ to the intestinal fluid, the administration of the probiotic bacteria maintained the levels of this cytokine similar to the basal data, at difference of the *S *group, which showed a significant decrease of IFNγ concentration after infection (Figure 2B). IFNγ (+) cells also increased in healthy mice given probiotic bacteria in both inductor and effector sites of the immune response compared to the untreated control group (Figure 1B and Table 1). This is consistent with previous reports where the administration of probiotic suspensions or fermented milks was associated with increased number of IFNγ (+) cells in the small intestine of mice [4,18]. Recent findings revealed an inhibitory effect of IFNγ on neutrophils trafficking and pro-inflammatory Th17 cells differentiation [19-21]. According to this observation, the increased levels of this cytokine in *Lc-S-Lc *group could be correlated with the reduced spread of *Salmonella *and the lower inflammation of small intestinal tissues observed previously [7]. IL-6 was analyzed because promotes both B cell maturation [22] and pro-inflammatory activity [23]. It was observed that 7 days after *Salmonella *challenge, the production of this cytokine in the small intestine tissues was significantly increased in the three infected groups compared with the untreated control (C), and 10 days post-challenge, only the group *Lc-S-Lc *maintained a number of IL-6 (+) cells higher than both control groups (C and *S*, Figure 1C). However, in the mice fed continuously with the probiotic (*Lc-S-Lc *group), the IL-6 release into the intestinal lumen remained stable 7 and 10 days post-infection. In contrast, the infection control group (*S*) significantly increased IL-6 secretion during all the experiment, compared with basal data (Figure 2C). These results showed that probiotic administration can down regulate the release of IL-6 but maintain increased production of this cytokine in the intestine which could be used by the host if it is required.

According with the results obtained for the mentioned cytokines, IL-10 was studied as an anti-inflammatory cytokine and similar to IL-6 is required to maintain the IgA (+) B cell population [24,25]. In our work, 7 days post challenge the number of IL-10 (+) cells was significantly higher in infected mice that received probiotic administration than in mice from *S *group, (Figure 1D). As regard to this cytokine release, the concentration of IL-10 in the intestinal fluid was significantly decreased in the infected control group (*S*) throughout the study, while in mice from *Lc-S *group the significant decrease was observed 10 days post infection. At day 7 post-challenge, IL-10 release of *Lc-S-Lc *group was lower than absolute control (group C) and *Lc *group, but restored at day 10 post-challenge. These results highlight the importance of continuous probiotic administration in the modulation of the immune response (Figure 2D).

Previous results obtained in our group suggested that probiotic administration modulates the cytokine profile, mainly in the cells from the innate immune response through TLRs stimulation [4,26]. According to this, and considering the differences observed for the cytokines, we analyzed the expression of TLRs in immune and epithelial cells of the small intestine in our infection model.

TLR2 was studied due this receptor recognize the peptidoglycan which is the principal component of the Gram+ bacteria such as *Lactobacillus *genus. Our results showed a significant increase of TLR2 (+) cells in the small intestine of healthy mice that received *L. casei *CRL 431 compared to the untreated control (Figure 3A) and significant increases were also observed, only for 7 days post infection, in the mice given continuously the probiotic bacteria (*Lc-S-Lc *group) compared to the infection control (*S *group). This result agrees with other findings describing a similar effect induced by two *Lactobacillus *strains, *L. rhamnosus *GG and *L. plantarum *BFE 1685, which enhanced TLR2 in vitro using human intestinal cells [10]. We consider that the probiotic strain stimulates the TLR2 not only to increase the signals to produce cytokines, but also to increase the epithelial barrier because it was demonstrated TLR2 activation have an important role in enhancing trans-epithelial resistance to invading bacteria [27]. Another receptor analyzed was TLR4, which recognizes the LPS present in the cell wall of the Gram(-) bacteria [28]. It is known that TLR4 plays a significant role in the host defences against *Salmonella *infection *in vivo *[29-31]. In our model, *L. casei *CRL 431 administration to healthy mice increased the number of TLR4 (+) cells compared to the untreated control, which could be used as a surveillance mechanism against pathogen bacteria such as *Salmonella*. Recent findings suggest that the activation of this receptor initiates an innate immune response leading to the induction of pro-inflammatory mediators, to increase TLR2 expression, and to reduce its own expression, which leads to the recruitment of inflammatory cells and the initiation of the appropriate responses in the spleen leading control of the bacterial multiplication [29,32]. This is consistent with the results obtained in our study where the enhancement of TLR4 was accompanied of increased number of TLR2 (+) cells previous and post infection (Figure 3). The early increase in the expression of TLR4 could be related with the decrease of the severity of the infection observed in the treated groups where the bacterial growth in the spleen and the liver decreased faster than in the infection control [7].

TLR5 was evaluated because flagellated bacteria, including *E. coli *and *Salmonella*, can interact with TLR5 to induce activation of pro-inflammatory gene programs for host protection [33-35]. In the present work, we observed that probiotic administration increased TLR5 (+) cells after *Salmonella *infection in both groups that received the probiotic strain for 7 days post challenge compared to untreated mice (C, Figure 3C). This finding agrees with other study where two lactobacilli were able to increase the cell surface expression of TLR5 in HT29 cells to respond to *S*. Typhimurium [10]. In our model, this receptor could be also implicated in the protective effect of *L. casei *CRL 431 against *S*. Typhimurium infection.

Finally, in our study, it was observed that *L. casei *CRL 431 oral administration increased TLR9 expression in healthy mice (Figure 3D). Seven days post infection, the increase of TLR9 (+) cells was observed in both groups of mice given probiotic bacteria (*Lc-S *and *Lc-S-Lc*), but not in the infection control (*S *group), comparing with the untreated control group (C). This finding agrees with several works which affirm that CpG-TLR9 interaction can improve the resistance of normal adult mice to a variety of bacterial, viral and parasitic pathogens [36-38], including increased resistance to oral challenge with *S*. Typhimurium. TLR9 signalling is also required to mediate an anti-inflammatory effect induced by probiotics, in a mouse colitis model [39].

## Conclusions

The results of the present work demonstrated the importance of *L. casei *CRL 431 continuous administration, before and after *S*. Typhimurium infection, to maintain the mechanisms of protection against this pathogen. *L. casei *CRL 431 administration before infection maintained the innate immune system in alert state, through modulated expression of TLRs and cytokine signals in the effector and inductor site of the gut immune system, which could be related with the protection against *S*. Typhimurium observed in a previous report. The results from the present work show that once established the disease, the continuous *L. casei *CRL 431 administration protected the host mainly modulating the inflammatory response against the enteropathogen in both effector and inductor sites of the gut. This preliminary study shows some of the immune mechanisms implicated in the protective effect of *L. casei *CRL 431 againts *S*. Typhimurium infection. More studies should be performed to validate the use of this probiotic strain in the prevention and as a complement to treatments in the defense against salmonellosis.

The cellular populations involved in the cytokine production and how TLRs activate the different signals and the transcriptional factors for cytokine production are currently under study.

## Methods

### Animals and experimental groups

Five-week-old BALB/c mice weighting 22-26 g were obtained from the closed random bred colony maintained at CERELA (Centro de Referencia para Lactobacilos, San Miguel de Tucumán, Argentina). The assays were performed using 3 experimental groups to assess the effect of the preventive or continuous probiotic administration against *S*. Typhimurium infection comparing with the infection control group (*S*). The same number of female and male mice was distributed in all the groups.

For the study of the mechanisms involved in the preventive effect, mice received *L. casei *CRL 431 for 7 consecutive days before challenge with the enteropathogen (*Lc*-*S *group). For the effect of the continuous probiotic administration, mice were administered *L. casei *CRL 431 during 7 days, challenged with the pathogen and then continued receiving *L. casei *CRL 431 post challenge (*Lc*-*S*-*Lc *group). Mice of the infection control group (*S*) did not receive special feeding and were challenged with *S*. Typhimurium. Additionally, two control groups without infection (healthy mice) were analyzed: a group of mice received *L. casei *CRL 431 (*Lc *group), and the other group did not received special feeding (untreated control group, C). Mice were euthanized and the samples were collected after 7 days (the day of the infection) for *Lc *and C groups, and 7 and/or 10 days post challenge (depending on the assay performed) for all the groups.

All animal protocols were pre-approved by the Animal Protection Committee of CERELA and all experiments complied with the current laws of Argentina.

### Bacterial strains

*L. casei *CRL 431 was obtained from the CERELA culture collection. Overnight cultures were grown at 37°C in sterile Mann-Rogosa-Sharp (MRS) broth (Britania, Buenos Aires, Argentina). The cells were harvested by centrifugation at 5 000*g *for 10 minutes, washed three times with fresh PBS and then resuspended in sterile 10% (vol/vol) non-fat milk. *L. casei *CRL 431 was administered to the mice in the drinking water to reach a concentration of 1 × 10^8 ^CFU/ml. This lactobacilli count was periodically controlled at the beginning and after 24 h of dilution in water (maintained in the same room where the mice are) to avoid modifications of more than 1 logarithmic unit.

*S*. Typhimurium strain was obtained from the Bacteriology Department of the Hospital del Niño Jesús (San Miguel de Tucumán, Argentina). An aliquot (200 μl) from an overnight culture was placed in 5 ml of sterile Brain Heart Infusion (BHI) broth (Britania, Buenos Aires, Argentina) and incubated during 4 hours. The concentration of *Salmonella *was adjusted to 1 × 10^8 ^CFU/ml in phosphate buffered saline (PBS). Each mouse was challenged with 100 μl of 1 × 10^8 ^CFU/ml of *S*. Typhimurium given by gavage. This dose was selected in our previous work because induce 50% of mice mortality [7].

### Isolation and culture of immune cells from Peyer's patches for cytokine determination

The protocol described by Galdeano and Perdigón [11] was used for the isolation of cells from Peyer's patches. The cells were isolated after 7 days of feeding for *Lc *and C groups and 7 days post *Salmonella *infection for all the challenged groups. The small intestine of each mouse was removed, washed and the Peyer's patches were excised in Hank's buffered salt solution (HBSS) containing 4% foetal bovine serum (FBS). The epithelium cells were separated with HBSS/FBS solution containing EDTA. The sediments were incubated with dispase/DNAse solution and the mononuclear cells were recovered. The cells were collected and washed with RPMI 1640 medium (Sigma, St. Louis, USA). The cells concentrations were adjusted in RPMI and cultured in a CO_2 _incubator. The culture supernatants were recovered after 4 h and 24 h for TNFα and after 24 h to analyze the levels of IFNγ and IL-10 using ELISA technique. BD OptEIA mouse cytokine ELISA sets from BD Bioscience (San Diego, USA) were used according manufacturer instructions. The results were expressed as concentration of each cytokine (pg/ml).

### Detection of cytokine producing cells isolated from Peyer's patches

Mononuclear cells were isolated from Peyer's patches as described above. 20 μl of each cell suspension (1 × 10^6^) was placed per well in special glass slides by triplicate. They were fixed 15 minutes with BD Pharmigen ICC Fixation Buffer. TNFα, IFNγ and IL-10 were determined by immunocytochemistry following the technique described by Dogi et al [40]. Briefly, the glass slides were incubated with a blocking solution of bovine serum albumin (BSA)/PBS, washed with PBS, and incubated with normal goat serum (Sigma, St. Louis, USA). The activity of the endogenous peroxidase was blocked with a peroxidase blocking reagent (Dako Cytomation, Inc., California, USA). The cells were then incubated with avidin and biotin blocking solutions (Avidin/biotin blocking kit, Vector laboratories, Inc., Burlingame, USA) to block endogenous avidin and biotin. The cells were incubated with rat anti-mouse TNFα, IFNγ or IL-10 (diluted in ICC cytokine buffer, PharMingen, B-D Biosciences, Canada), washed with PBS, and incubated with goat anti-rat polyclonal antibody conjugated with peroxidase (PharMingen, B-D Biosciences, Canada). Vectastain Elite ABC solution (Vector Labs, Burlingame, USA) was added to cells and incubated with a DAB kit (Vector Laboratories, Inc., Burlingame, USA). The results were obtained from two individual blind counts per each sample (by two different investigators) and were expressed as number of positive cells counted per 2 × 10^4 ^cells at 1 000X magnification.

### Determination of cytokine producing cells in the lamina propria of the small intestine

The small intestines were removed after 7 days of feeding (*Lc *and C groups), and 7 and 10 days post *Salmonella *challenge for all experimental groups, and processed following the technique described by Sainte-Marie for paraffin embedding [41]. Tissue sections (4 μm) from each mouse were used to analyze cytokine producing cells by an indirect immunofluorescence assay following the technique described previously [11]. The sections were incubated with a blocking solution of BSA/PBS, washed with PBS, and incubated with normal goat serum (Sigma, St. Louis, USA) to prevent non-specific staining. Rabbit anti-mouse TNFα, IFNγ, IL-10, and IL-6 (Peprotech, Inc. Rocky Hill, NJ, USA) polyclonal antibodies (diluted in saponin-PBS) were applied to the tissue sections for 105 min at room temperature (RT, 21°C). The sections were then treated 1 h with diluted goat anti-rabbit antibody conjugated with fluorescein isothiocyanate (FITC, Jackson Immuno Research, Labs. Inc. West Grove USA). The results were expressed as the number of fluorescent cells in 10 fields of vision as seen with 1 000X magnification using a fluorescent light microscope. The number of fluorescent cells was counted for two different investigators (by blind counts) three times to cover different portions of each sample.

### Determination of TNFα, IFNγ, IL-10 and IL-6 released in the small intestine fluid

Intestinal fluid from the small intestines of all the groups under study were collected with 1 ml of NaCl 0.85% at the same time points that the samples from intestinal tissues. The fluids were immediately centrifuged at 4 000*g *during 15 min at 4°C. The supernatants were recovered and stored at -20°C until cytokines determination by ELISA using the methodology previously described for cell culture supernatants. The results were expressed as concentration of each cytokine in the intestinal fluid (pg/ml).

### Immunofluorescence assays for determination of TLR2, TLR4, TLR5 and TLR9 positive cells on small intestine tissues

TLR2, TLR4, TLR5 and TLR9 positive cells were counted in the samples taken at the same time points used to determine the cytokine producing cells. Positive cells for each analyzed TLR were counted in the small intestine tissue (including lamina propria and epithelium or intraepithelial cells) for all the groups assayed. After deparaffinization and rehydration, paraffin sections were incubated with solution of 1% BSA for 30 min at room temperature and washed three times in PBS. Rat anti-mouse monoclonal TLR2 or TLR4 (eBioscience, USA) diluted 1:300, rabbit anti-mouse polyclonal TLR5 (Santa Cruz Biotechnology, INC) diluted 1:250 or TLR9 (eBioscience, USA) in a concentration of 0.5 μg/ml antibodies, were applied to the tissue sections for 105 min at room temperature. The slides were washed twice with PBS and incubated for 60 min with a dilution of FITC conjugated goat anti-rat (1:50) or goat anti-rabbit (1:100) antibody (Jackson Immuno Research Labs Inc.). The results were expressed as the number of fluorescent cells in ten fields of vision at 1 000X of magnification and they were obtained from two individual blind counts per each sample (by two different investigators).

### Statistical analysis

Each trial, test and control groups contained 10 animals. Three mice of each group were sacrificed for each sample taken. The experiments were repeated three times and all results (from the three trials) were analyzed together (N = 9). Statistical analyses were performed using MINITAB 14 software. A factorial experimental design (replicates - dietary regimen - time point) was used. Comparisons were accomplished by an ANOVA general linear model followed by a Tukey's post hoc test and p < 0.01 was considered significant. No significant differences between the three independent replicates were observed.

## Competing interests

The authors declare that they have no competing interests.

## Authors' contributions

NAC carried out the microbiological work and the animal studies. GP and AdMdL conceived of the study. NAC, AdMdL and GP designed the experiments. NAC performed the statistical analyses and prepared the figures. NAC and AdMdL wrote the draft of the manuscript. GP revised it for significant intellectual content. All authors read and approved the final version of the manuscript.
